# A Basic Strategy to Manage Global Health with Reference to Livestock Production in Asia

**DOI:** 10.4061/2011/328307

**Published:** 2011-10-31

**Authors:** David C. Hall, Quynh Ba Le

**Affiliations:** Department of Ecosystem and Public Health, Faculty of Veterinary Medicine, University of Calgary, Calgary, AB, Canada T2N 2Z6

## Abstract

Newly emerging infectious diseases (nEIDs) have increased rapidly presenting alarming challenges to global health. We argue that for effective management of global health a basic strategy should include at least three essential tactical forms: actions of a directly focused nature, institutional coordination, and disciplinary integration in approaches to health management. Each level of action is illustrated with examples from the livestock sector in Asia. No clear example of all three tactical forms in place can be found from developing countries where food security is a significant threat although Vietnam is developing a comprehensive strategy. Finally, an ecosystem health approach to global health management is advocated; such an approach moves away from the traditional single disciplinary approach. Stronger guidance is needed to direct ecohealth research and application in the management of global health.

## 1. Introduction

In the last two decades, as many countries have broadened their network of trading partners, moved quickly to adopt previously unimagined technologies, and hastened the pace of economic migration, it has become increasingly apparent that health too is a global matter that commands a global perspective. The severe acute respiratory syndrome (SARS) pandemic (There was and is still disagreement as to whether or not SARS was a pandemic. By the Center for Disease Control (CDC) standards, SARS was a pandemic (http://www.cdc.gov/ncidod/EID/vol10no11/04-0797_02.htm). The simplest WHO definition of a pandemic at the time was “a worldwide epidemic of a disease". Although the World Health Organization (WHO) did indeed declare that we had entered the early phases of a pandemic, the official declaration of the highest stage of a pandemic, Phase 6, was never made) in 2003 accentuated this point, as did the Influenza Type A/H1N1 outbreaks of 2009. But before one jumps to the erroneous conclusion that globalization is the new demon, we need to recall that there have long been global movements of disease with devastating consequences such as the smallpox epidemic brought upon the Aztecs by invading Spanish Conquistadors in the 1500s [1]. No doubt the infrastructural differences in today's world mean the risk of a pandemic is greater than 500 years ago but so too are our defenses, particularly in terms of scientific technologies, research and knowledge transfer capacity, and institutional coordination. This paper outlines a broad approach to framing a strategy for managing and improving global health. For a global health management strategy to be effective, we argue that tactics must take place in at least three essential forms: actions of a directly focused nature, institutional coordination, and disciplinary integration in approaches to health management. We illustrate each level of action with current examples from the livestock sector in Asia including village level interventions to increase food security in Bangladesh, an example from Vietnam of improved institutional integration and cooperation to control emerging infectious disease, and improved capacity of researchers and other health personnel to adopt an ecosystem approach to health management in Asia.

The complexities of social and ecological interactions are such that no single approach to managing health resources can adequately compensate for inequities that result in poor access to health care at the village level, unsustainable management of resources leading to the emergence of zoonotic disease, or unrealized institutional partnerships to manage regional health risks such as the recent threat of an Influenza Type A/H1N1 pandemic emerging from Southeast Asia and North America. Root problems may show themselves as poverty and hunger as a result of low incomes and lack of market access in remote villages in northern Bangladesh, lack of awareness or concern for environmental contamination resulting from factors (there are of course innumerable factors, but they include attitudes and preferences, sociocultural beliefs, and the cost of change) including low education or inappropriately distributed market power among stakeholders in livestock producing villages in Southeast Asia, or high institutional transaction costs that prevent collaboration or integration of high-level health management strategies in Vietnam. But regardless of the nature and complexity of the root problems, responses to the challenges of addressing health issues of concern to the global population require improved knowledge, expanded capacity, and determined collaboration of individual stakeholders including end users, researchers, and local-to-regional governments and health related institutions.

## 2. Newly Emerging Infectious Diseases, Changes in the Global Human Condition, and the Health of Small-Scale Livestock Producers

Newly emerging infectious diseases (nEIDs) such as SARS, H5N1, and Nipah virus encephalitis are health issues with global impact. They seem to emerge without apparent warning, they spread with great speed, and they impose severe threats to global health, ranging from human to animal to economic health. Over the last ten years, these and other nEIDs have appeared in either Bangladesh or Vietnam and sometimes both countries, resulting in significant impact on the health and livelihoods of communities through increased morbidity and mortality of humans and livestock. Furthermore, hundreds of millions of dollars of economic damage has been caused from disruptions in local and regional trade, as well as enforced cessation of livestock production. The impact has been most severe among the rural poor—those who are able least to invoke mitigation strategies to the risks posed by global health challenges.

Changes in human socioeconomic conditions include job loss or abandonment leading to poverty and hunger, rapid population growth and uncontrolled urbanization, movements of political, economic, and environmental refugees, exploitative behaviors and policies, and new contacts between wild fauna and humans and their livestock have altered the emergence and transmission of nEIDs [2]. The impact and implications of these changes on the health of small-scale livestock producers can be severe, most particularly where zoonotic diseases are involved. For example, all 93 human cases of highly pathogenic avian influenza (HPAI) in Vietnam, from 2003 to 2005, 42 of which were fatal, had a history of exposure to sick poultry [3].

Contributing to these conditions is the remarkably rapid expansion of livestock ownership in low-income countries. In Vietnam for example, livestock ownership has expanded rapidly in the last decade with more than 80% of small-scale rural households owning some form of livestock [4, 5]. More than 50% of rural livelihoods depend on livestock for nutrition and cash income in both Vietnam [6] and Bangladesh [7], and because the majority of small-scale livestock keepers are women, livestock also generates employment for women and offers them economic decision making power. The importance of this last point is often not fully appreciated with respect to decisions for the welfare of the household. When women who raise livestock are able to decide how and when the income is spent on the household, then they have control over varying and balancing food inputs for children and infants, increasing food security and improving the health of their family.

The important contributions of livestock to health and economic well being are considered as part of a global veterinary mission, identified by the Steinfeld of the Food and Agricultural Organization (FAO) of the United Nations as a strategy for improving livelihoods in developing countries [8]. Livestock not only provide farmers with a way to increase investment but also promoting security and health of the household. Randolph et al. [9] have outlined the same points, noting the important role of livestock in improving human nutrition and health and reducing and preventing poverty.

Clearly livestock are important in poverty alleviation and health maintenance, but the threat of losing that source of income or possible infection of the livestock keepers themselves increases with each new outbreak of HPAI or other zoonotic disease.

## 3. Risky Farming Practices with Focus on Livestock Production

In the Chars region of north and north-west Bangladesh, a high proportion of villagers are landless, live in extreme poverty, and face annual cycles of flooding resulting in annual hunger and malnutrition. This is a major precipitating factor for health problems among the rural poor, leading to low birth weight, delayed physical and mental development, and susceptibility to infections [10]. The use of livestock and horticulture has been accepted as a suitable intervention by some villagers in the Chars area [11] with initial success. Extending this intervention, integrated agriculture (the incorporation of sustainable crop and livestock practices) in an ecohealth (ecohealth can be defined as the field of study and practice that uses systemic and participatory approaches to understanding and promoting health and well being in the context of complex socialecological interactions) framework has been introduced as an additional option to address these extreme problems [12]. Preliminary results indicate that household income increased 60–70% when farmers invested in dairy cattle, allowing for economic decisions to be made (and increasing the role of women in making those decisions) regarding purchases of food, clothing, and medicine, and allowing for children to attend local schooling. The result has improved nutrition and economic power of inhabitants, reducing poverty and hunger and increasing child health and employment (For a more thorough example of integrated health management in an ecohealth framework employed to reduce risk of disease, refer to Joshi and associated papers: Durga Datt Joshi and Minu Sharma. An Urban Ecosystem Health Approach to Make A Cleaner City and Better Health in Kathmandu, Nepal. National Zoonoses and Food Hygiene Research Centre (NZFHRC), Kathmandu, Nepal-Paper presented in World Congress of Public Health, August 18–26, 2006, Brazil.)

Despite this step towards eradicating poverty in the Chars region of Bangladesh, major constraints remain. Productivity of cattle is low due to poor genetics and weak knowledge of management skills, there is limited integration of environmental sustainability options, and coliform bacteria from livestock pose a health hazard to humans who live in close contact with their livestock day and night. The latter is of more than local concern for the reasons described already; recent changes in *Escherichia coli* have been noted globally, generating apprehension over the possibility of the emergence from the livestock sector of “super-bugs” resistant to available antibiotics and generating world-wide concerns for health of animals and humans. This is also one of the reasons, consistent with observations of the World Health Organization, that poor livestock keepers are more likely to bear the brunt of the impact of zoonotic disease [13].

For now these constraints and concerns in the Chars of the Jamuna River in north central Bangladesh are being addressed locally at the village community level through training programmes, technical interventions, and improved access to health and agricultural services. This then is a good example of the first of the three essential forms of tactics in a global health management strategy (and surely the most common): action at the grass roots level, or perhaps more politely termed directly targeted interventions. Regrettably, the other two essential forms of tactics are in relatively early stages in Bangladesh. Disciplinary integration in approaches to health management is rare, and institutional coordination is the exception rather than the rule, largely due to financial, educational, and to some extent political barriers at the level of institutions. Early progress has been made in the form of a National Livestock Policy that advocates these concepts with an action plan although this policy is yet to be ratified by the Government of Bangladesh [14]. This is an important step towards policy support for an integrated approach that will include improved access to health and agricultural services and targeted technical interventions intended to address poverty while leaving intact options for small-scale backyard livestock keeping.

Without a research environment and appropriate policy that supports wider development and adoption of an integrated ecosystem approach to health management in low income countries where options are limited for rapid mobilization and change, the use of the grass roots level tactic is likely to remain dominant for many years to come. This does not speak well of mitigating the contributing factors and risks of inequities in human health; furthermore, it is a tactic without teeth for managing global health. 

Vietnam has its share of directly targeted interventions in the livestock sector as well although as we shall see a more broadly thinking strategy is developing in the livestock sectors of Vietnam to embrace the second and third tactics of institutional coordination and disciplinary integration. Nevertheless, Pfeiffer et al. [15] has noted that in Vietnam “agri-livestock farming systems involving domestic water birds and rice production in river delta areas are important for the maintenance and spread of infection.” Restructuring of the livestock sector in Vietnam has already begun, as have the other tactics suggested in this paper.

## 4. Moving from Single Disciplinary to Institutionally Coordinated Approaches

While we argue that directly targeted interventions are necessary, they require the support of other broader tactics to be of relative importance in a wider based approach to managing global health. Livestock policy responses addressing poverty alleviation have tended to focus on intensive short-term interventions targeting single nEIDs (e.g., HPAI control policies in SEAsia), generally within the confines of solitary disciplines rather than long-term sustainable solutions which integrate truly transdisciplinary approaches. In Vietnam, emerging infectious diseases (EIDs) such as avian influenza and the association of livestock with EIDs have generated considerable control-based activities, particularly at the farm level [16].

For highly pathogenic avian influenza (HPAI) and occasionally for pig diseases including swine influenza (H1N1), options have included broad-scale depopulation, targeted culling, movement control, segregation of species of livestock, and vaccination. Knowledge of coordinated large-scale response programmes has increased, and Vietnamese government agencies have developed well-planned and- implemented programmes to respond to nEIDs such as HPAI. Evidence from Vietnam and Thailand indicates that while such programmes may reduce the risk of EIDs after farms have become infected, they do not address pre-infection strategies [17]. Furthermore, they do little to address the longer-term economic and household nutrition needs of poor rural households that are likely to be engaging in small-scale raising of livestock.

It has been recognized by the Government of Vietnam and supporters of pro-poor policies that, in order to maintain access to small-scale livestock which provides income and maintains food security while reducing the precipitating factors of infectious disease, it is necessary to consider restructuring livestock-keeping methods and strategies [18]. Such restructuring methods would not remove livestock but rather recommend methods by which livestock might be raised such that the risks of EIDs are greatly reduced. This could include integrated agriculture rather than focusing on single species of plants and animals, managing water resources to reduce contact of animal species with each other and with humans, developing alternative methods of livestock housing to prevent the transmission of pathogenic organisms, and following simple steps when raising livestock to ensure the health of those livestock and subsequently the health of the families around those livestock. Proposed changes could take place throughout the market chain, not only at the level of the household. 

Implementation of some of these changes, which would require transdisciplinary understanding and collaboration, has been tried on a small scale with rather limited success. Visits to participating communes in Vietnam in 2009 by the authors revealed that less than 10–15% of households had adopted more than two of eight main recommendations [19]; no formal study has been published from study of these communes to examine the reasons for low adoption and implementation or the impact on health of women and children. Although the use of a multidisciplinary team is a giant step in a positive direction, we believe that part of the reason for low adoption may well be the focus on a single species outcome rather than an integrated outcome that balances environmental sustainability with community partnership and free choice of economic activities. In other words, results from adopting integrated agriculture and ecosystem approaches to health management have to be driven by basic understanding of the transdisciplinary issues and by free choice to engage in economic activities.

At the institutional level, Vietnam has made significant progress in this area with the development of the Government of Vietnam-United Nations Joint Program (JP) to fight Highly Pathogenic Avian Influenza (HPAI). The JP is a solid example of multi-institutional effort to address nEIDs and national health inequities using the resources of numerous government ministries and international nongovernment agencies. The JP initially addressed the immediate emergency support needed to control the outbreaks of HPAI that started in 2005. The programme has continued to a new phase in which design and implementation has included international coordination/technical assistance, communication, agriculture (livestock production and animal health), and human health involving the Ministry of Agriculture and Rural Development (MARD), the Ministry of Health (MoH), the United Nations Development Program (UNDP), the United Nations Children's Fund (UNICEF), the Food and Agriculture Organization of the UN (FAO), and the World Health Organization (WHO). The overall objective of the program is “to reduce the health risk to humans from avian influenza by controlling the disease at source in domestic poultry, by detecting and responding promptly to human cases, and by preparing for the medical consequences of a human pandemic.” More specifically, the program aims at reduced risk of a global pandemic of HPAI emanating from Viet Nam and enhanced national and local capacity to manage outbreaks of diseases of epidemic potential caused by human and animal pathogens.

Some of the key findings from the midterm evaluation of this program were that it has had impact on coordinating within and across the UN agencies and Ministries of the Government of Vietnam, and improved coordination between implementing agencies of the JP has resulted in a more holistic approach to solving a critical health issue for Vietnam and the region. However, the concept of sustainable ecosystem health to prevent emerging infectious disease is present in bits and pieces of activities throughout the JP (e.g., surveillance, training of community animal and human health workers, communication, vaccination, and restructuring) but is not a major pillar of understanding behind many of the activities (and was not intended to be). It was also recommended that although an ecohealth philosophy is evidently emerging in the JP strategy, wider knowledge of an ecohealth approach is needed, particularly at lower administrative levels, in order to appreciate the integrated roles of managing the interfaces of animals, humans, and the environment. Most importantly, this must be directed at nEIDs in general, rather than (e.g.,) HPAI or Influenza Type A/H1N1. Expanding this concept, as a broader theme is current being considered in the development of the new phase of the JP.

## 5. Building Ecohealth Capacity in Asia

Finally we examine an example of development of the capacity of researchers and ecohealth professionals to adopt an ecosystem approach to health management in Asia, the third essential form of tactic needed for a global health management strategy to be effective. The nongovernment organization Veterinarians Without Borders/Vétérinaires Sans Frontières—Canada (VWB/VSF-Canada) is implementing the Building Ecohealth Capacity in Asia (BECA) project, a three-year initiative to build ecohealth capacity in six Southeast Asian countries: Cambodia, China, Indonesia, Laos, Thailand, and Vietnam [20]. The initiative, funded primarily by the International Development Research Centre (IDRC) and Australian Agency for International Development (AusAID), aims to build knowledge and skills in ecohealth in order to help local ecohealth practitioners identify and reduce factors that contribute to emerging infectious diseases in the region.

A large part of the project focuses on transfer of knowledge to high-level researchers working in lead research institutions in Southeast Asia, as well as government and not-for-profit institutions from the same region working in health and agriculture. Together these partners will help drive the project through network development and through the creation of new ecohealth training and knowledge transfer opportunities. This research project will investigate the processes involved in building the capacity for research and application of ecosystem approaches to health management among researchers, development practitioners, and policy makers in Southeast Asia. The research will also investigate the methodologies and tools which contribute to effective capacity building in ecosystem approaches. Existing regional networks such as the Asian Partnership for Emerging Infectious Disease Research (APEIR) have already begun defining the kinds of research agendas and personal relationships that are necessary to make an ecosystem approach effective. 

The BECA research project will enhance opportunities for these and newer regional networks to identify and build capacity through the research activities of this project. The working hypothesis of this project is that bringing actors together from different countries and institutions with an array of experience and expertise in the prevention of EIDs, public health, and health promotion will enable participants to investigate and respond more effectively to complex ecohealth issues, with a particular focus on EIDs. This work will contribute in the longer term to an effective network of expertise in ecosystem approaches to managing health, contributing to ecohealth skills, and knowledge development in the region including input to ecohealth policy formulation. The target audiences of this project include academic researchers, government staff, and extension personnel who are and will be involved in promoting integration and application of ecohealth approaches in activities which directly benefit communities in need.

In brief, there are four main outputs expected from an initiative of this kind:

1. identification and promotion of methodologies for developing and measuring impact of sustainable ecosystem approaches to health;
2. promotion of research and application of trans-boundary sustainable ecosystem approaches to health through informing and influencing local and regional policy formulation;
3. development of a research and training network led by high-level researchers, decision makers, and public health workers in the region to build capacity in ecosystem approaches to health, addressing in particular the control of EIDs in South and Southeast Asia;
4. provision of a process-oriented evaluation and consolidation of adaptive and effective learning and teaching tools for building ecosystem approaches to health.

The project is past the first six months of its activities and has engaged with more than fifty participants through focused discussions and exercises at workshop sessions. While this approach serves to bring similar minded researchers and ecohealth practitioners together, it remains to be seen how well the principles and skills imparted in these training sessions will be incorporated into local and regional activities, programmes, and policies directed at reducing nEIDs. Thus far, at least we can report that several of the network participants have incorporated the knowledge learned from this project into research proposals and at least one policy position working paper addressing, for example, the reemergence of rabies in Bali, Indonesia after decades of being rabies free.

This is not the only project in the region that aims to build capacity in an ecohealth approach to management of health. APEIR is leading research in avian influenza in Southeast Asia that to a large extent relies on integrated approaches to research and application, and IDRC and the International Livestock Research Institute are directing related projects with different outputs. These various initiatives have in common the fact that they all embrace an ecohealth approach in some manner and address similar long-term goals of reduction of poverty and improvement of livelihoods through livestock.

## 6. Stronger Evidence Needed for Ecohealth Approaches Integrating Livestock Production

An integrated ecosystem approach to health management is advocated in this paper and in the projects described above as an approach to controlling and preventing nEIDs. Each of the three essential forms of tactics that we have identified would be important for implementing results of ecohealth research, but the groundwork in ecohealth research is still in its formative stages. We are calling for more research directed to ecosystem approaches to health management in order to inform development of sustainable solutions that improve the health and livelihoods of communities. Such approaches examine animal and human health as well as environmental and socioeconomic health and the complex interrelationships between these four dimensions. 

Understanding these complexities is critical to exploring the causes of nEIDs and to proposing recommendations for sustainable changes, particularly with respect to methods for raising livestock in a sustainable integrated agricultural system in low income countries such as Bangladesh and Vietnam, barriers to adoption and implementation of such methods, the role of health policy at local, national, and regional levels in promoting and supporting recommended programmes, evaluation of the impact on production, and impact on reducing the precipitating factors of nEIDs. In the particular case of an ecohealth approach that includes livestock within an integrated agriculture system, the goal of improving livelihoods through increased food security will be achieved through healthier livestock and humans, leading to improvements in productivity, increased incomes, and a greater degree of control over economic decision making at the household and community level. Ultimately this means less risk of health inequities in agricultural communities in poor countries and a reduction in global poverty and hunger.

## 7. Conclusions

In this paper, we have presented a broad straightforward approach to framing strategies for managing and improving global health. The three essential forms of tactics that we present—actions of a directly focused nature, institutional coordination, and disciplinary integration in approaches to health management—have been illustrated with examples from the livestock sector in Asia. Evidence from developing countries indicates that this is indeed the general philosophy that has been adopted by governments, research institutions, the nongovernment sector, and other stakeholders working in poverty alleviation through livestock production and integrated agricultural development. It is difficult to find a clear example of all three forms of tactics in use, but the examples presented from Vietnam perhaps present the model in place that comes closest to illustrating this broad approach. 

Village level interventions will continue to be important in addressing food security and global health, particularly where communities take a strong role in managing their own resources. Without research, we clearly would be lacking in guidance as to important new directions for knowledge generation and transfer related to correcting health inequities. And finally, the example of the Government of Vietnam-UN Joint Programme to fight Highly Pathogenic Avian Influenza illustrates that cooperation between large complex institutions in addressing a common goal is possible. The latter is not without its challenges, but the JP has been successful enough to warrant its use as a model for a broader One UN concept in which governments, international institutions, and development agencies bridge the sometimes enormous gaps between their institutions to share resources and engage in transdisciplinary problem solving in order to build sustainable solutions to global resources through global health management.

We have also advocated for an ecosystem approach to health management, which is viewed as a more holistic approach to problem solving in global health by examining not just the biological mechanisms of disease but also the complexities of social-ecological interactions. Applied health research, particularly as it relates to zoonotic and nEIDs, does seem to be gravitating in the direction of ecohealth applications. However, clear guidance from ecohealth research is needed to identify primary areas of investigation that will yield sustainable solutions of high impact on poverty, livestock and human health, and environmental management.
